# After In Vitro Digestion, Jackfruit Flake Affords Protection against Acrylamide-Induced Oxidative Damage

**DOI:** 10.3390/molecules24183322

**Published:** 2019-09-12

**Authors:** Daofeng Qu, Chu Liu, Mengxue Jiang, Lifang Feng, Yuewen Chen, Jianzhong Han

**Affiliations:** 1 School of food science and biotechnology, Zhejiang Gongshang University, Hangzhou 310018, China; daofeng@mail.zjgsu.edu.cn (D.Q.); fenglifang705@126.com (L.F.); chenyw@zjsu.edu.cn (Y.C.); 2 Food Safety Key Laboratory of Zhejiang Province, School of Food Science and Biotechnology, Zhejiang Gongshang University, Hangzhou 310035, China

**Keywords:** jackfruit flake, in vitro digestion, polyphenol, oxidative stress, high-content analysis

## Abstract

Some studies have demonstrated that acrylamide (AA) was correlated with oxidative stress, resulting in physical damage. The jackfruit flake was an immature pulp that contained a high level of antioxidant activity. This study aimed to assess the defensive efficacy of jackfruit flake in AA-induced oxidative stress before and after simulated gastrointestinal digestion. Our results indicate that the total polyphenol content of Jackfruit flake digest (Digestive products of jackfruit flake after gastrointestinal, JFG) was diminished; however, JFG had raised the relative antioxidant capacity compared to Jackfruit flake extract (JFE). Additionally, the results of High Performance Liquid Chromatography-Mass Spectrometry (HPLC-MS) implied that a proportion of compounds were degraded/converted into other unknown and/or undetected metabolites. Further, by high content analysis (HCA) techniques, JFG markedly reduced cytotoxicity and excessive production of reactive oxygen species (ROS) in cells, thereby alleviating mitochondrial disorders. In this study, it may be converted active compounds after digestion that had preferable protective effects against AA-induced oxidative damage.

## 1. Introduction

As a potential carcinogen, acrylamide (AA), threatening human health, is widely found in foods. Some studies have reported that AA mainly derives from the Maillard reaction of aspartic acid and reducing sugar at high temperatures [1]. As far as we know, there is widespread exposure to high amounts of AA from heated food in human daily life. In the last years, most researchers showed that AA has strong carcinogenicity, neurotoxicity, and genotoxicity [2]. In recent years, some studies have monitored that AA is easily absorbed by intestinal cells, increasing the accumulation of intracellular reactive oxygen.

The scientific name of jackfruit is *Artocarpus heterophyllus* Lam., belonging to the Moraceae family. Jackfruit flake is immature pulp, most of which is used as waste for goat and pig feed [3]. Recently, jackfruit has gained increasing popularity in the world, and the consumption is so large that the flake is discarded too much. Moreover, most studies have shown that the plant is rich in phytochemicals, such as flavonoids, phenolic acids, and carotenoids [4], which may play an important role in the prevention of several chronic diseases. Jackfruit flake has been proven to have strong antioxidant properties [5]. Meanwhile, the jackfruit pulp has an anti-proliferative effect on the M12.C3.F6 cell, preventing active free radicals in biomolecules [6]. It is widely recognized that food can only work in the body through the gastrointestinal tract, due to the existence of an enzyme that hydrolyzes macromolecular substances in food matrices. Furthermore, studying the antioxidant properties of polyphenolic chemicals in food cannot reflect the real situation, ignoring the chemical changes in the gastrointestinal digestion process. At present, although many studies have observed that an in vitro gastrointestinal digestion model has been used to simulate the digestion of food in the human gastrointestinal tract, most of them use a static stomach, which lacks the changes of pH in the human stomach [7]. 

High content analysis (HCA) technology consists of automated microscopy and image analysis and has emerged as a tool of cell biology, which provides a novel way to evaluate a variety of cell biological parameters combining fluorescent dyes. In cytotoxicity studies, HCA has already been demonstrated to be a kind of analytical means to assess cytotoxicity responses [8].

In this study, the human digestive tract environment was reflected by a model of the digestive process consisting of a simulated dynamic stomach and small intestine. Then after in vitro gastrointestinal digestion, the individual polyphenolic compound in food matrices was identified by HPLC-MS, which screened undegraded polyphenols. Further, we verified the change of intracellular oxidative stress, mitochondrial membrane potential, and mitochondrial permeability by using fluorescent dye and HCA technology. 

## 2. Results

### 2.1. Total Phenolic Content and Total Antioxidant Capacity

The impact of JFE, Digestive products of jackfruit flake in the stomach (JFG_0_), and JFG on total phenolic content (TPC) and total antioxidant capacity (AC) are shown in Table 1. When compared to JFE, phenolic contents were reduced stepwise from gastric to intestinal digestion. The overall tread of reducing antioxidant capacity was similar to total phenolics. As shown by the data, the TPC and AC of JFG were significantly decreased by 68.12% and 53.93% (*p* < 0.05), respectively, compared with JFE. However, the relative antioxidant capacity (AC/TPC) of JFG was two-fifths higher than JFE.

### 2.2. Qualitative Identification of Compounds

**Figure 1:**
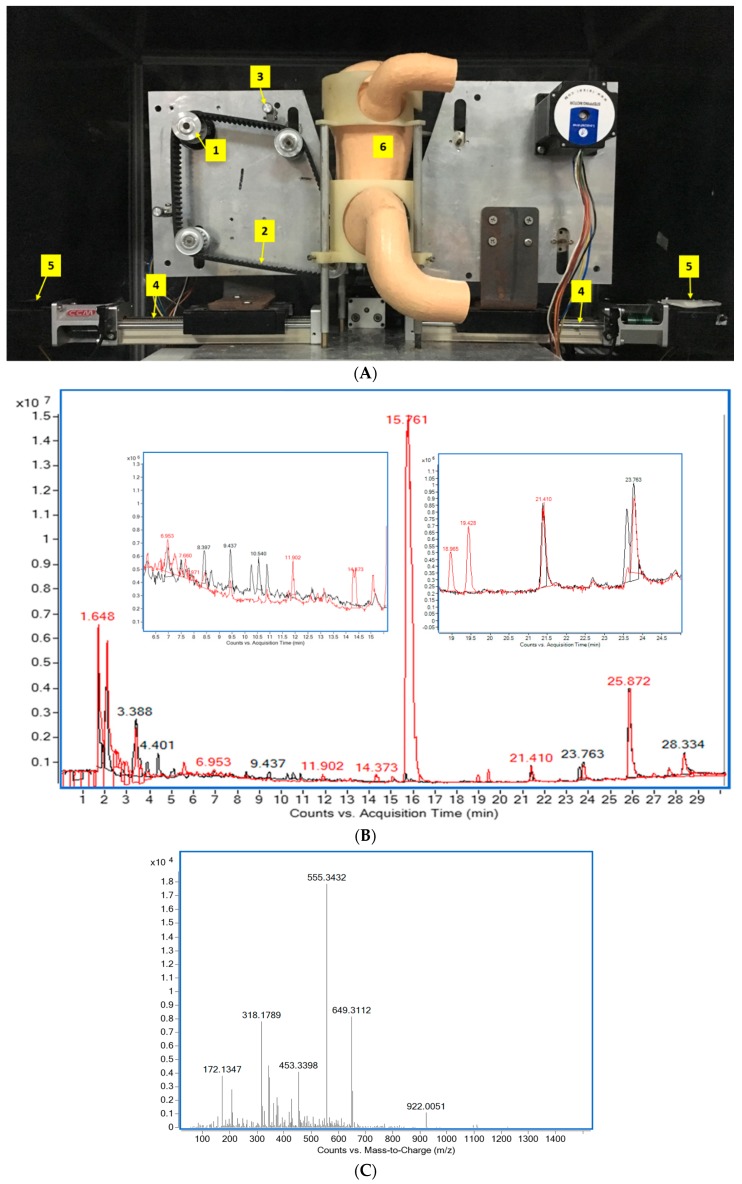

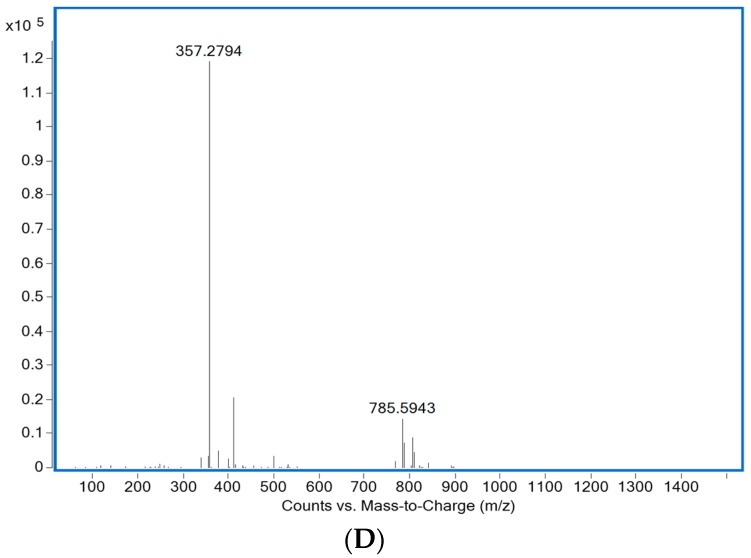
(**A**) In vitro dynamic stomach model: the numbers 1–6 respectively represent stepper motor, timing belt, compression roller, timing belt guide, screw motor, and silicone stomach. (**B**) Representative chromatograms highlighting the compounds characterized before and after the digestion of Jackfruit flake: The chromatogram was obtained using the positive ion mode. (**C**) Mass spectrum of compound (**6**) in positive mode. (**D**) Mass spectrum of compound (**11**) in positive mode.

To test whether the polyphenols in the jackfruit flake change before and after digestion, the phenolic compositions of JFE and JFG were identified by HPLC-MS. The compounds detected by time-of-flight mass spectrometer (TOF) can be used to obtain the molecular formula based on the exact molecular weight as shown in Table 2 and Figure 1B. The peaks of the main 21 compounds are listed based on the peak areas of the individual phytochemicals. The possible identities of these 21 compounds were determined by the data in the scientific literature and the available real standards: (3) prenyl-7-hydroxy [9]; (4) marmesin isomer [9]; (6) caffeic acid derivative [10,11]; (7) apigenin-6-C-glucosyl-8 carabinoside [12]; (8) citric acid derivative [11], (10) ferulic acid-*O*-hexoside [11], (11) feruloylglucoside [10,11,13], (12) caffeoylquinic acid [10,11], (13) skullcapflavon [12], (14) neochlorogenic [10], (16) gartanin [14], (18) dihydroxybenzoic acid malonyl hexoside [15], and (20) *p*-coumaric acid-*O*-hexoside [10,11,13]. The compounds were identified by data from the experiments and the scientific literature. Compound (**6**) (t_R_ =7.66 min) displayed a [M + H]^+^ ion at *m*/*z* 555; according to [10,11], the compound may be considered to be a caffeic acid derivative (Figure 1C), and compound (**11**) (t_R_ = 18.965 min), [M + H]^+^ at *m*/*z* 357, classified as hydroxycinnamic acid, may be feruloylglucoside (Figure 1D). The summarized results showed that a proportion of compounds in jackfruit flake were degraded/converted into other unknown and/or undetected metabolites after the in vitro digestion.

### 2.3. Effect of Samples before and after Digestion on AA-Induced Cytotoxicity

**Figure 2:**
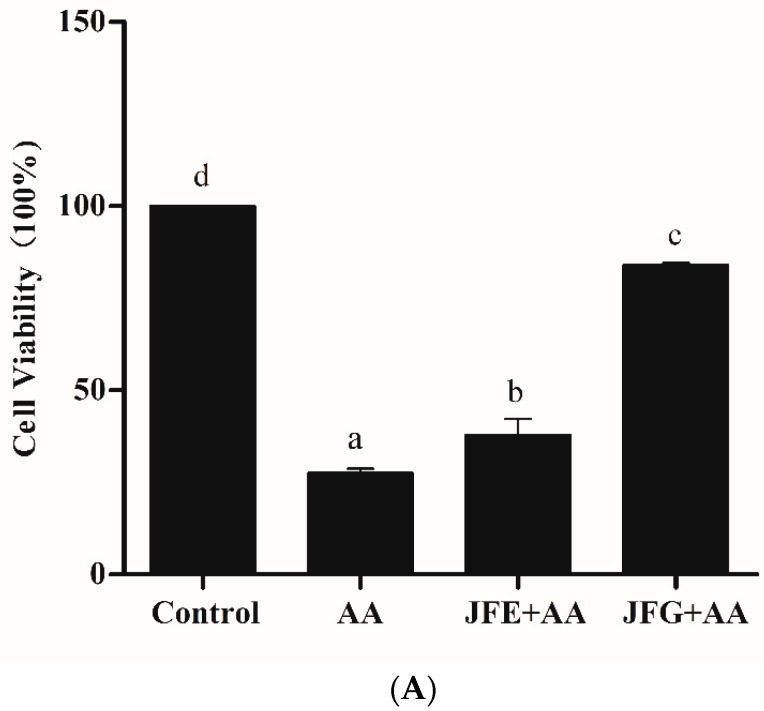

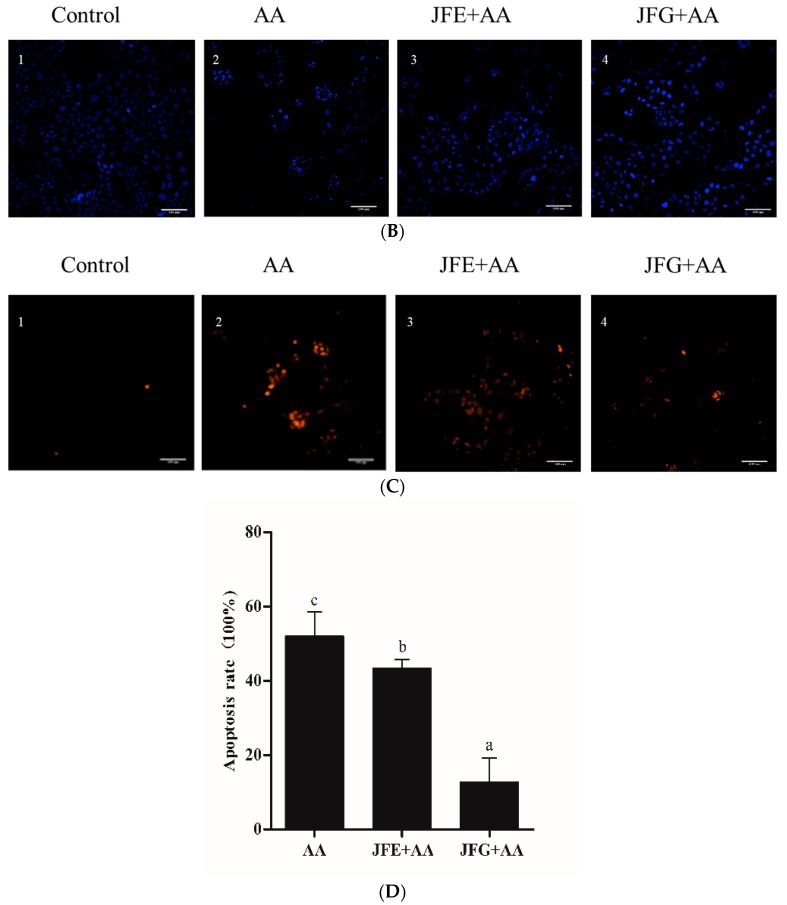
Effects of JFE and JFG on AA-induced Caco-2 toxicity. (**A**) Caco-2 cells were treated with 2 g/L AA for 24 h, with or without 2 mg/mL JFE or JFG for 24 h, cell viability was measured by 3-(4,5-Dimethylthiazol-2-yl)-2,5-diphenyltetrazolium bromide (MTT). (**B**,**C**) Caco-2 cells were treated with 2 g/L AA for 2 h, with or without 2 mg/mL JFE or 2 mg/mL JFG for 24 h, and then stained with Hoechst 33342 and PI and photographed using high-content analysis (HCA). Scale bars = 100 μm. (**D**) The JFE groups and JFG groups on apoptosis rate of Caco-2 cells for 24 h. The data was expressed as mean percent (mean ± standard deviations) and different letters in the bars within each group represent statistically significant differences (*p* < 0.05).

According to the above results, the protective effect of Jackfruit flake on AA-induced cytotoxicity before and after digestion was evaluated, and AA was used to induce cytotoxicity in Caco-2 cells. As shown in Figure 2A, when compared with control groups (cell viability set to 100%), the cell viability of AA groups was reduced to 27.51% ± 1.17% (*p* < 0.05). However, compared with AA groups, JFE and JFG groups significantly increased by 37.97% ± 4.19% and 84.03% ± 0.67% (*p* < 0.05), respectively.

Based on these phenomena, we used Hoechst 33342 and Propidium Iodide (PI) fluorescent dye and further explored the apoptosis of AA-induced cytotoxicity in Caco-2 cells in JFE and JFG. In Figure 2B,C, the mean fluorescence intensity of AA groups was significantly increased compared with control groups. Additionally, the mean fluorescence intensity of JFE and JFG was weaker than that of the AA groups, especially JFG. In Figure 2D, compared with control groups, the apoptotic rates of AA groups were 51.99% ± 6.40% (*p* < 0.05). In contrast, the apoptosis rate of JFG decreased to 12.72% ± 6.48% (*p* < 0.05). The result suggested JFG was able to effectively ameliorate AA induced toxicity.

### 2.4. The Effects of Samples before and after Digestion in AA-Induced ROS Levels

**Figure 3:**
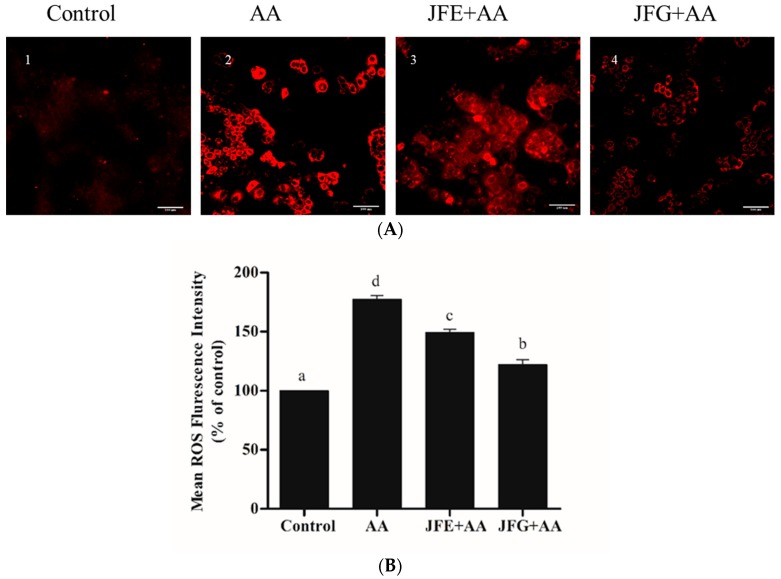
Deep red reagent which was located in the cytoplasm. (**A**) Caco-2 cells were treated with 2 g/L AA for 2 h, with or without 2 mg/mL JFE or JFG for 24 h, and photographed using HCA. Scale bars = 100 μm (**B**). The mean ROS fluorescence intensity of AA groups, JFE groups, and JFG groups, and the control groups (set to 100%). The data was expressed as mean percent (mean ± standard deviations), and different letters in the bars within each group represent statistically significant differences (*p* < 0.05).

As shown in Figure 3B, the fluorescence intensity of AA groups increased to 177.5% ± 2.43% (*p* < 0.05) compared with control groups (the mean fluorescence intensity was 100%). Moreover, JFE and JFG groups significantly reduced the mean fluorescence intensity to 149.3% ± 0.86% and 122.2% ± 1.70% (*p* < 0.05), respectively, indicating that JFE and JFG had protective effects on AA-induced oxidative stress in Caco-2 cells. JFG provided more effective protection.

### 2.5. Samples before and after Digestion Attenuated AA-Induced Oxidative Damage to Mitochondrial Membrane Potential

**Figure 4:**
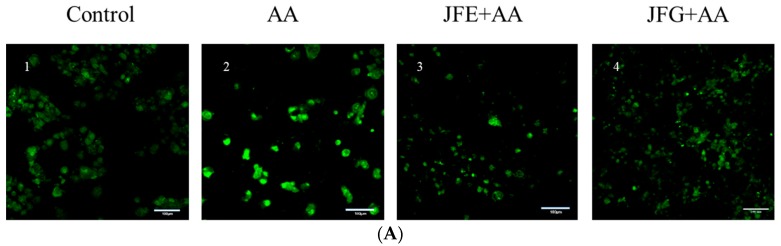

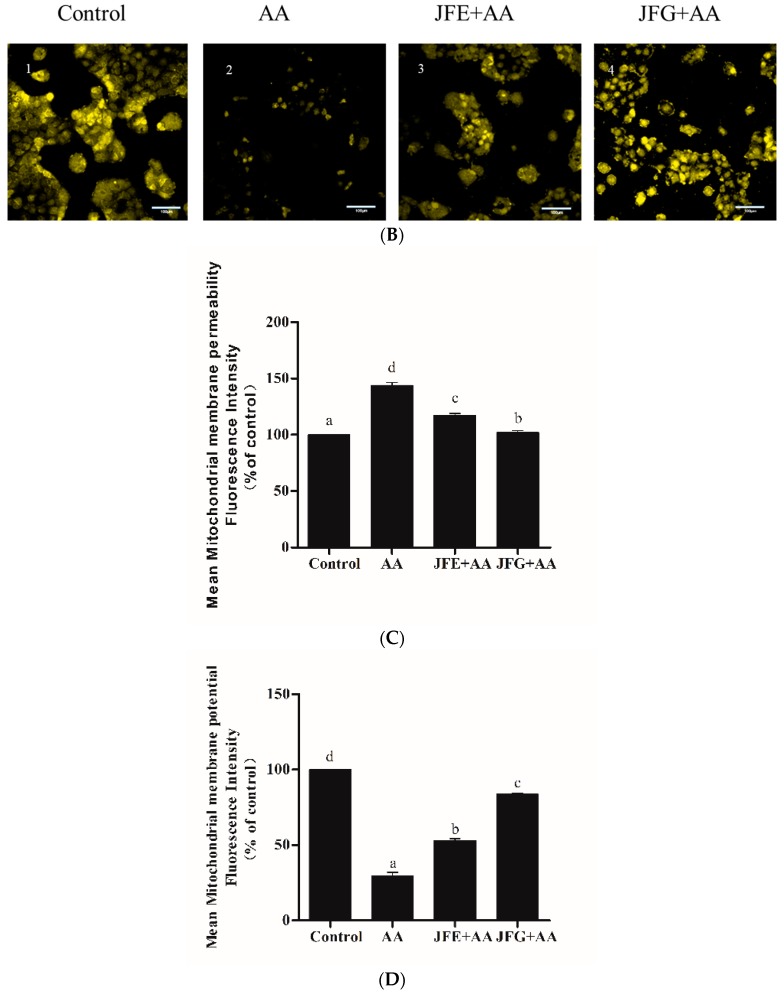
Effects of JFE and JFG on AA-induced Caco-2 mitochondrial permeability and mitochondrial membrane potential. (**A**,**B**) Caco-2 cells were treated with 2 g/L AA for 2 h, with or without 2 mg/mL JFE or JFG for 24 h, and photographed using HCA. Scale bars = 100 μm. (**C**,**D**) The mean mitochondrial permeability and mitochondrial membrane potential fluorescence intensity of AA groups, JFE groups, and JFG groups were determined by Image-IT^®^ DEAD Green^TM^ viability stain, and the control groups were set to 100%. The data was expressed as mean percent (mean ± standard deviations), and different letters in the bars within each group represent statistically significant differences (*p* < 0.05).

Mitochondria are important plants for cellular aerobic respiration and oxidative phosphorylation, and their health is closely related to the amount of intracellular ROS. In Figure 4A,C, the mean fluorescence intensity of the mitochondrial membrane potential was significantly decreased to 29.65% ± 0.61% (*p* < 0.05), and the mitochondrial membrane permeability was 143.97% ± 1.41% (*p* < 0.05) in AA groups, compared with the control groups. To the contrary, the mitochondrial membrane potential mean fluorescence intensities of the JFE and JFG groups were significantly increased by 59.01% ± 0.78% and 83.66% ± 0.72% (*p* < 0.05) of the control groups, respectively, and the mitochondrial membrane permeability mean fluorescence intensity of the JFE and JFG groups were 117.05% ± 1.93% and 102.13% ± 1.64% (*p* < 0.05), respectively. 

## 3. Discussion

AA was rapidly absorbed by intestinal cells. The protective effects of JFE and JFG on oxidative stress of AA-induced cytotoxicity in Caco-2 cells have been confirmed, and JFG enhanced the effect.

The polyphenol content and antioxidant capacity of Jackfruit flake were degraded after digestion in the stomach and intestine. In the gastric phase, polyphenol contents were slightly reduced, which was possibly related to acidic conditions during in dynamic gastric digestion. It has been reported [16] that a pH of 2.0 in the gastric phase affected the stability of low molecular weight polyphenols in *Arbutus*. In addition, under acidic conditions, some polyphenols may form a gel with soluble fiber resulting in partial degradation of polyphenol contents after gastric digestion [17]. From the above experiments, it was concluded that the polyphenol contents of Jackfruit flake were greatly reduced after gastrointestinal digestion, which was consistent with the results for apples [18]. Loss of polyphenol compounds after intestinal digestion may be linked to the presence of pancreatic enzymes and bile [19], and the food was transiently transferred from a pH of 2.0 to a pH of 7.0 in the intestine, which affected polyphenol degradation. It simulated the true digestion of the human stomach in an in vitro dynamic gastric model, and the pH constantly changed during this process, unlike the static stomach [20]. The reduction of polyphenol compounds in the gastrointestinal digestion process was also associated with the matrix of the food itself. The release of polyphenol compounds in persimmon powder matrix depended on the variety and type of bioactive compounds, and it was mainly relevant to dietary fiber contents and various interactions between dietary components and bioactive compounds, resulting in the reduction of bio acceptability [18]. The experiment determined that the antioxidant properties of JFG were much inferior to JFE, which may be the reason for the low contents of polyphenols.

However, results from another study suggest that polyphenol content and antioxidant capacity in samples increased after gastrointestinal digestion [21]. This may be related to the matrix of the food itself, the structure and type of phytochemicals, and different digestion conditions (time, temperature, pH) of in vitro gastrointestinal digestion, especially, structural changes of released compounds of food matrix during digestion, glycosylation and esterification with other compounds, which made a difference in results [22]. The results of LC-MS showed that phenolic acid was most abundant after gastrointestinal digestion. It was possible to judge that the polyphenol substance in the food has changed and exhibits that a certain substance interacts with other compounds in the gastrointestinal digestion process to form another substance. For example, bayberry digesta had more phenolic acids than extracts [23], neochlorogenic acid in chokeberry was reduced by 28%, and the chlorogenic acid was increased by 24% [24]. Therefore, it favorably explained that the relative antioxidant capacity of JFG was stronger than JFE.

Therefore, the changes in the nuclei stained by Hoechst 33342 were monitored to determine apoptosis. The number of cells in the JFG group was significantly higher than in JFE and AA groups. Moreover, the apoptotic rate was decreased, which may indicate an addition in feruloylglucoside. Because ferulic acid did not degrade and undergo intestinal transit in the gastric acid environment [25], it had a strong antioxidant capacity and promoted cell proliferation. It was further detected that JFG strongly reduced associated oxidative damage. High levels of ROS accumulation led to apoptosis and mitochondrial dysfunction because mitochondria were the main source of intracellular ROS. In this study, it was notable that an increased peak and a new peak were identified, including caffeic acid derivative and feruloylglucoside, which may be a reason for the protection of Caco-2 cells from oxidative stress through inhibiting ROS generation and enhance antioxidant ability. Moreover, it is also a possible reason for multiple polyphenols to exert a synergistic effect to resist the damage of reactive oxygen species. Studies by [26] demonstrated that the reconstituted mixture of apple juice (containing rutin, phloridzin, chlorogenic acid, caffeic acid, and epicatechin) effectively reduced ROS levels and protected the gut from reactive oxygen. Therefore, experimental data showed various polyphenols in JFG lowered mitochondrial membrane permeability and improved mitochondrial membrane potential.

We have demonstrated that the digested jackfruit flake has enhanced protection against oxidative damage caused by acrylamide. Additionally, we need a deeper understanding of the underlying mechanism to reveal a new strategy for the prevention of oxidative stress-caused diseases.

## 4. Materials and Methods

### 4.1. Materials and Reagents

Jackfruits were obtained from Hainan Province, China. Acrylamide was purchased from Aladin. Pepsin (2000 U/mL), pancreatin (800 U/mL), bile, dulbecco’s modified eagle medium (DMEM) culture media, and Folin–Ciocalteu’s phenol reagent were purchased from Sigma Chemical (St Louis, MO, USA). Caco-2 was obtained from the Shanghai Institute of Cell Biology at the Chinese Academy of Sciences (Hangzhou, China). Hoechst 33342 and PI were purchased from Beyotime Institute of Biotechnology (Shanghai, China). HCS Mitochondrial Health Kit and Cell ROX^®^ Oxidative Stress Reagent were purchased by Thermo Scientific (San Jose, CA, USA). All other reagents were of analytical grade.

### 4.2. Sample Preparation 

The flake was separated from the Jackfruits. Then they were freeze-dried for 24 h, pulverized into a fine powder to ensure uniformity, and stored at −4 °C.

### 4.3. Extraction of the Flake

The flake dried powder (1 g) was extracted with 30 mL of methanol/water (90/10, *v*/*v*) for 25 min at 100 rpm with ultrasonic-assisted extraction, and subsequently, centrifuged for 10 min at 4000 rpm and 4 °C. The residue was re-extracted, and then the supernatants were collected. After the prepared extracts were evaporated in a rotary evaporator at 37 °C under vacuum, the remaining phenolic concentrate was dissolved by methanol solution (90%, *v*/*v*), so that the final volume of 10 mL was obtained to get the final extracts which were referred to as JFE, and stored at −80 °C for further investigation. 

### 4.4. Simulated Gastrointestinal Digestion of Jackfruit Flake 

7.5 g of flake powder was mixed with 52.5 mL simulated salivary fluid (SSF) and 0.375 mL CaCl_2_·2H_2_O (44.1 g/L) to simulate oral digestion. As shown in Figure 1A, in the dynamic gastric digestion stage with a simulated human gastric digestion device, a mixture of 135 mL simulated gastric fluid (SGF), 90 μL CaCl_2_·2H_2_O, 1.11 g of pepsin (2000 U/mL), and the above-mentioned oral digesta was added in a silicone stomach and the pH automatically adjusted to 2 with HCl (1 M) in the device. Meanwhile, the temperature of the silicone stomach was maintained at 37 °C by thermostat. After 2 hours, the digesta (JFG_0_) were removed from the bottom of the stomach, stored at 20 °C until further analysis. Following the intestinal digestion stage, the pH of the gastric digesta was adjusted to 7.0 with NaOH (1 M). Thereafter, to the processed gastric digesta was added a mixture which contained simulated intestinal fluid (SIF), pancreatin (100 U/mL), bile (10 mM), and 90 μL CaCl_2_·2H_2_O and incubated at 37 °C for 2 h in a shaking water bath. After intestinal digestion, the intestinal digesta (JFG) was collected and stored at −80 °C for further investigation.

### 4.5. Characterization of Total Polyphenols and Antioxidant Capacity

#### 4.5.1. Determination of Total Phenolic Content

The Folin–Ciocalteu assay was used to determine the total phenolic content, according to article [27]. Briefly, JFE, JFG_0_, and JFG solution were mixed with 100 μL Folin–Ciocalteu reagent and incubated for 5 min. After a final volume of 1 mL with Na_2_CO_3_ solution (15%) was added to the mixture, the reaction took place for 2 h. Then 200 μL of the reaction solution was sequentially added to the plate, and the absorbance was measured at 760 nm. The total phenolic content was expressed by the gallic acid equivalent.

#### 4.5.2. Determination of Total Antioxidant Capacity

The method [28] was slightly modified. The total antioxidant capacity was measured with the Ferric ion reducing antioxidant power assays (FRAP). Briefly, a standard curve was constructed using iron (II) sulfate solution (range 0.4–2.0 mM with *n* = 5 concentrations), and the result was expressed as 1 μmol Fe (II) per 1 g fresh weight.

#### 4.5.3. Identification of Phenolic Compounds by HPLC-MS

Phenolic characteristics were identified using an Agilent 1200 HPLC (Shimadzu, Japan) equipped with a C18 column and TOF-MS system. The condition was carried out according to article [5]. JFE and JFG were filtered through a 0.22 μm membrane. The injection volume was 20 μL. Using the peak area, the content of the identified compounds in JFE and JFG was compared. 

### 4.6. Cell Culture

Caco-2 cells were cultured in DMEM medium which contained 10% fatal bovine serun (FBS, Gibco) and 100 U/mL of penicillin and streptomycin, and cultures were placed in a cell incubator with 5% CO_2_ at 37 °C.

### 4.7. Protective Effect against Acrylamide-induced Caco-2 Cell Oxidative Damage

#### 4.7.1. Cell Viability Assay

The tetrazolium salt (MTT) assay was used to measure cell viability. For the assay, Caco-2 cells (1 × 10^4^ cells/ml) were incubated in 96-well plates overnight, while moving the medium and adding JFE or JFG. Prior to this, cells were treated with 2 g/L AA in the presence or absence of JFE or JFG (2 mg/mL) for 24 h. After incubation with 0.5 mg/mL MTT for 4 h, dimethyl sulfoxide (DMSO) solution was added to the mediums. The optical density (OD) at 490 nm was detected using a microplate reader. Blank wells were set (blank culture solution, no cells added).

#### 4.7.2. Detection of Apoptosis

By double staining with Hoechst 33342 and PI, changes in apoptosis were assessed. In brief, after treatment, cells were incubated with 10 μg/mL Hoechst 33342 and PI at 37 °C for 20 min. The staining solution was removed, and the cells were washed three times with Phosphate Buffered Saline (PBS), photographed, and analyzed with HCA.

#### 4.7.3. Detection of Intracellular ROS Levels 

In short, after treatment, cells were incubated with 10 μg/mL of Cell ROX^®^ deep red reagent (Thermo Fisher Scientific, USA) at 37 °C for 30 min. Then the staining solution was washed with PBS and immediately measured using HCA.

#### 4.7.4. Detection of Mitochondrial Membrane Potential and Mitochondrial Membrane Permeability

The mitochondrial membrane potential and mitochondrial membrane permeability were monitored. In short, after treatment, cells were incubated with 10 μg/mL of Image-IT^®^ DEAD Green^TM^ (Thermo Fisher Scientific, USA) viability stain or Mito Health stain at 37 °C for 30 min. Then the staining solution was washed with PBS and immediately measured using HCA.

### 4.8. Statistical Analysis

All data were expressed as the mean ± standard deviation (SD) of at least three independent experiments. Using Origin 8.5 software (Northampton, MA, USA, Origin Lab), bar graphs were drawn and fluorescence intensity was calculated by HCA. When *p* < 0.05, differences were considered to be significant.

## Figures and Tables

**Table 1:** Total phenolic content (TPC), antioxidant capacity (AC), and ratio of antioxidant capacity per total phenolic content (AC/TPC) of extract and digesta of Jackfruit flake.

	Unit	JFE	JFG_0_	JFG
Total phenolic content (TPC)	mg GAE/mL	2.089 ± 0.13 a	1.665 ± 0.06 b	0.6660 ± 0.04 c
Antioxidant capacity (AC)	μmol Fe^2+^/mL	0.622 ± 0.02 a	0.5644 ± 0.02 b	0.3050 ± 0.02 c
AC/TPC	%	29.70%	33.90%	45.80%

Values are reported as means ± SD (*n* = 3), different letters in the same line indicate significant differences (*p* < 0.05) between treatments.

**Table 2:** Identification of indicated phenolic components from Jackfruit flake.

Peak	RT(min)	Molecular Weight	Possible Compounds
Increase			
1	1.648	131.1125	Unknown
2	2.063	165.1037	Unknown
3	2.43	230.17	Prenyl-7-hydroxy
4	2.669	246.1297	Marmesin isomer
5	6.964	211.1187	Unknown
6	7.66	554.3432	Caffeic acid derivative
7	11.902	564.3926	Apigenin-6-C-glucosyl-8 carabinoside
8	14.373	406.2784	Citric acid derivative
9	15.761	816.7375	Unknown
10	18.965	356.2823	Ferulic acid-*O*-hexoside
11	19.428	356.2794	Feruloylglucoside
Decrease			
12	4.401	354.0991	Caffeoylquinic acid
13	5.089	376.1074	Skullcapflavon
14	8.397	353.1739	Neochlorogenic
15	9.437	451.1758	Unknown
16	10.54	396.1404	Gartanin
17	23.763	278.1612	Unknown
No significant change			
18	25.853	403.2346	Dihydroxybenzoic acid malonyl hexoside
19	27.667	256.2659	Unknown
20	21.41	326.0776	p-Coumaric acid-*O*-hexoside
21	28.334	282.2782	Unknown

Increase means that the peak area of compound was significant increased after in vitro digestion, on the contrary, decrease represents that the peak area of compound was decreased after in vitro digestion.
